# Supplementary material for: Effect of MnxOy Nanoparticles Stabilized with Methionine on Germination of Barley Seeds (Hordeum vulgare L.)
Source: Nanomaterials (Basel). 2023 May 8;13(9):1577. doi: 10.3390/nano13091577 (PMC10180524; doi:10.3390/nano13091577)
Supplement: Supplementary file 1 [file nanomaterials-13-01577-s001.zip › nanomaterials-2313547-supplementary.pdf]

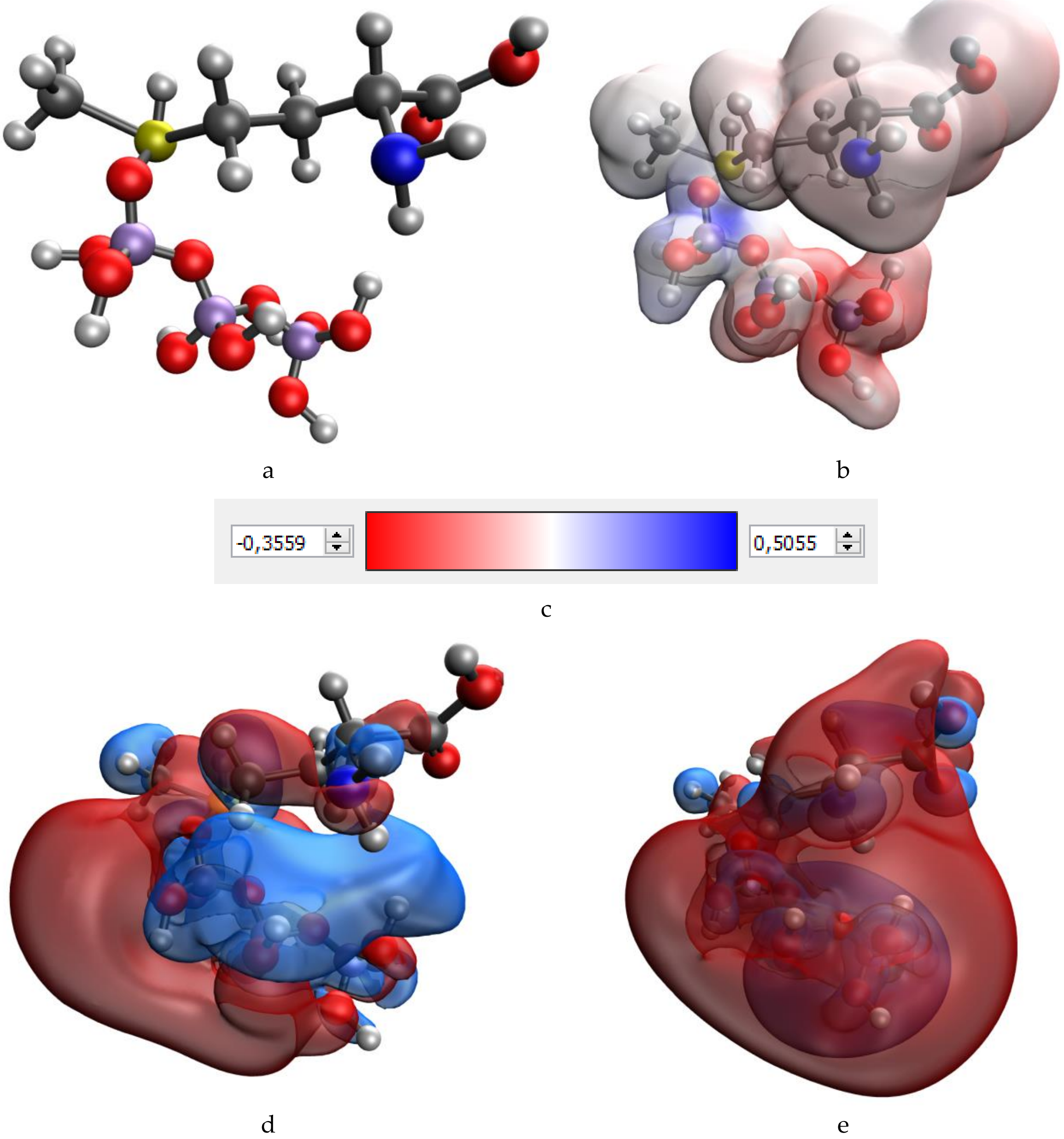

Figure S1. Interaction of the oxygen of the sulfo group of methionine sulfoxide and the hydroxo group of the extreme manganese atom of MnO_2: model of the molecular complex (a), electron density distribution (b), electron density distribution gradient (c), HOMO molecular orbital (d), LUMO molecular orbital (e)

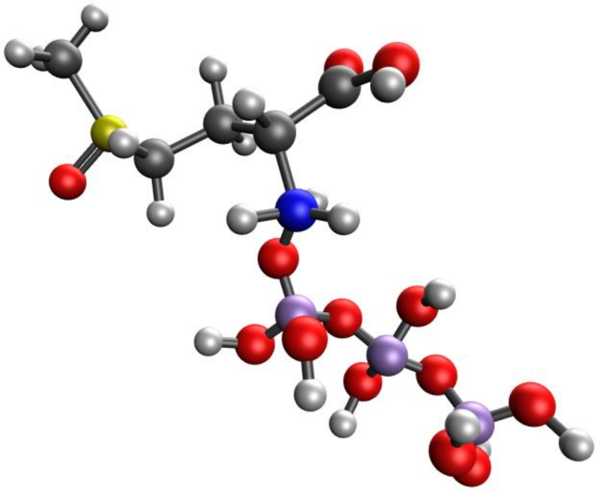

a

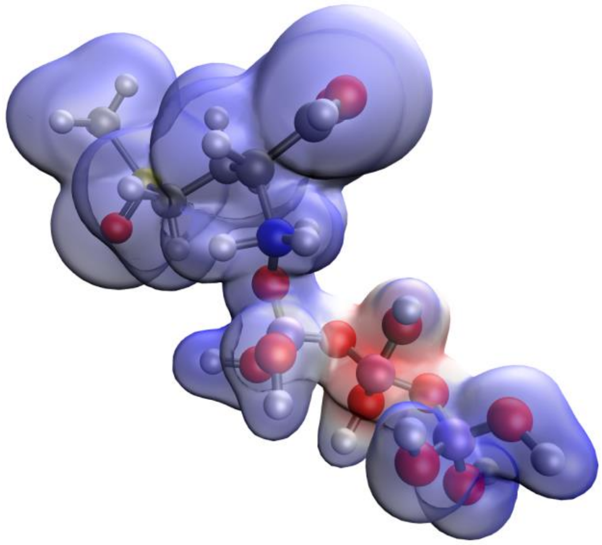

b

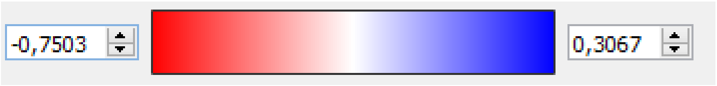

c

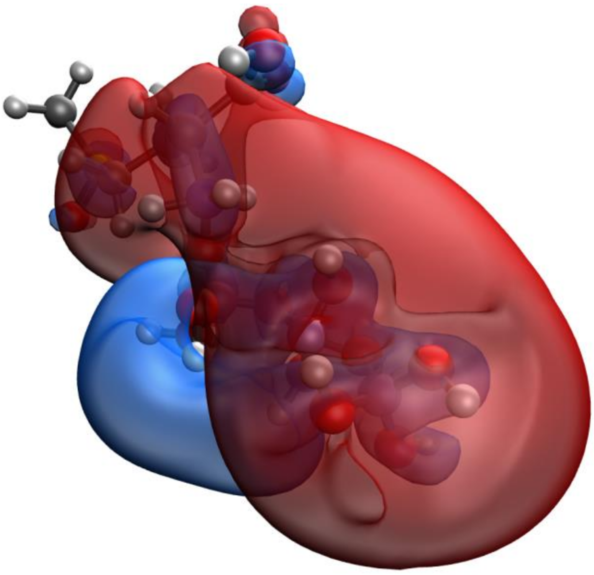

d

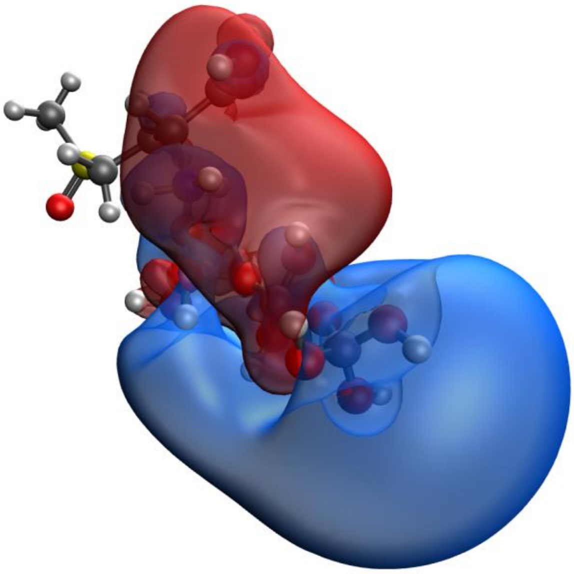

e

Figure S2. Interaction of the amino group of methionine sulfoxide and the hydroxo group of the extreme manganese atom of MnO_2: molecular complex model (a), electron density distribution (b), electron density distribution gradient (c), HOMO molecular orbital (d), LUMO molecular orbital (e)

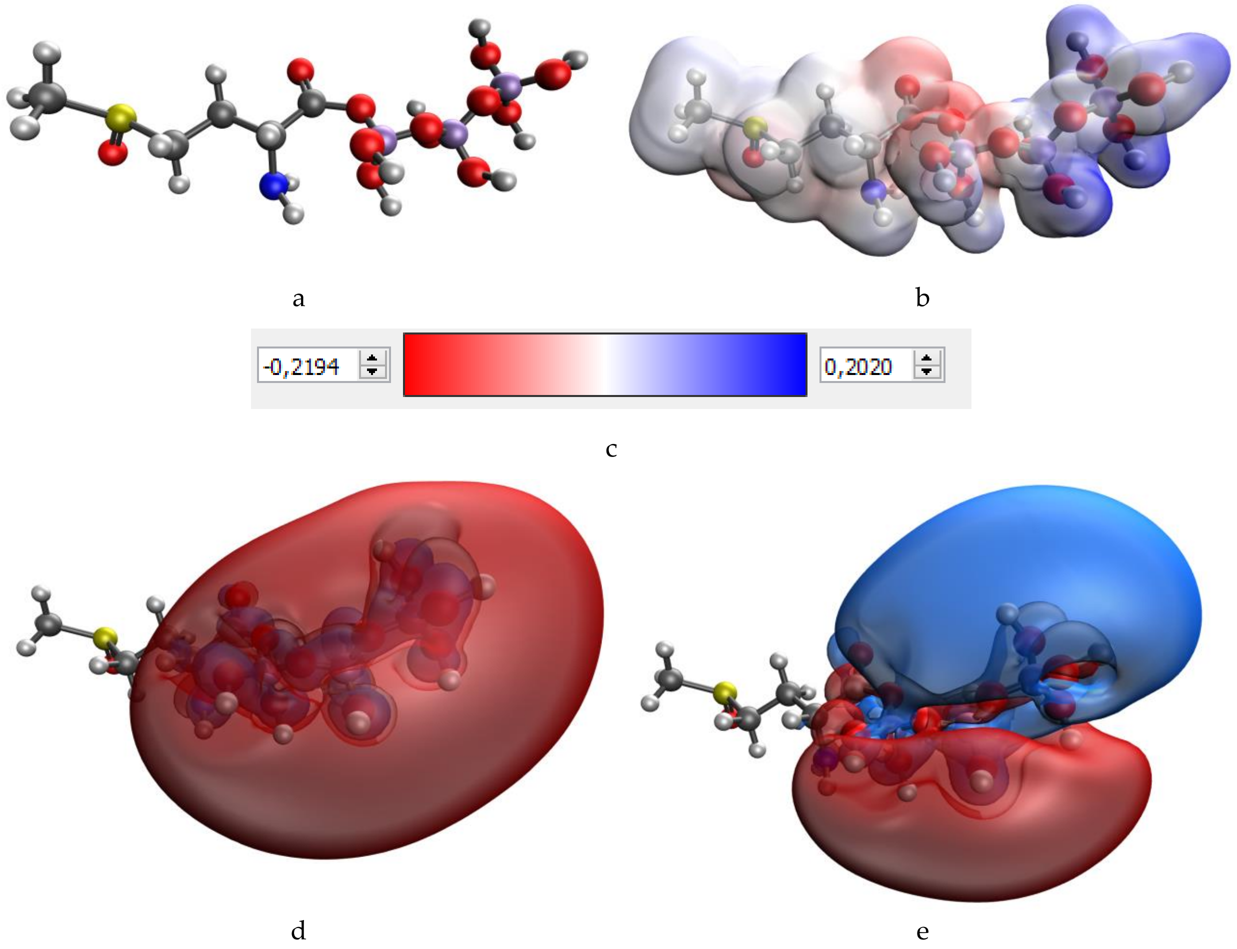

Figure S3. Interaction of the carboxyl group of methionine sulfoxide and the hydroxo group of the extreme manganese atom of MnO_2: model of the molecular complex (a), electron density distribution (b), electron density distribution gradient (c), HOMO molecular orbital (d), LUMO molecular orbital (e)

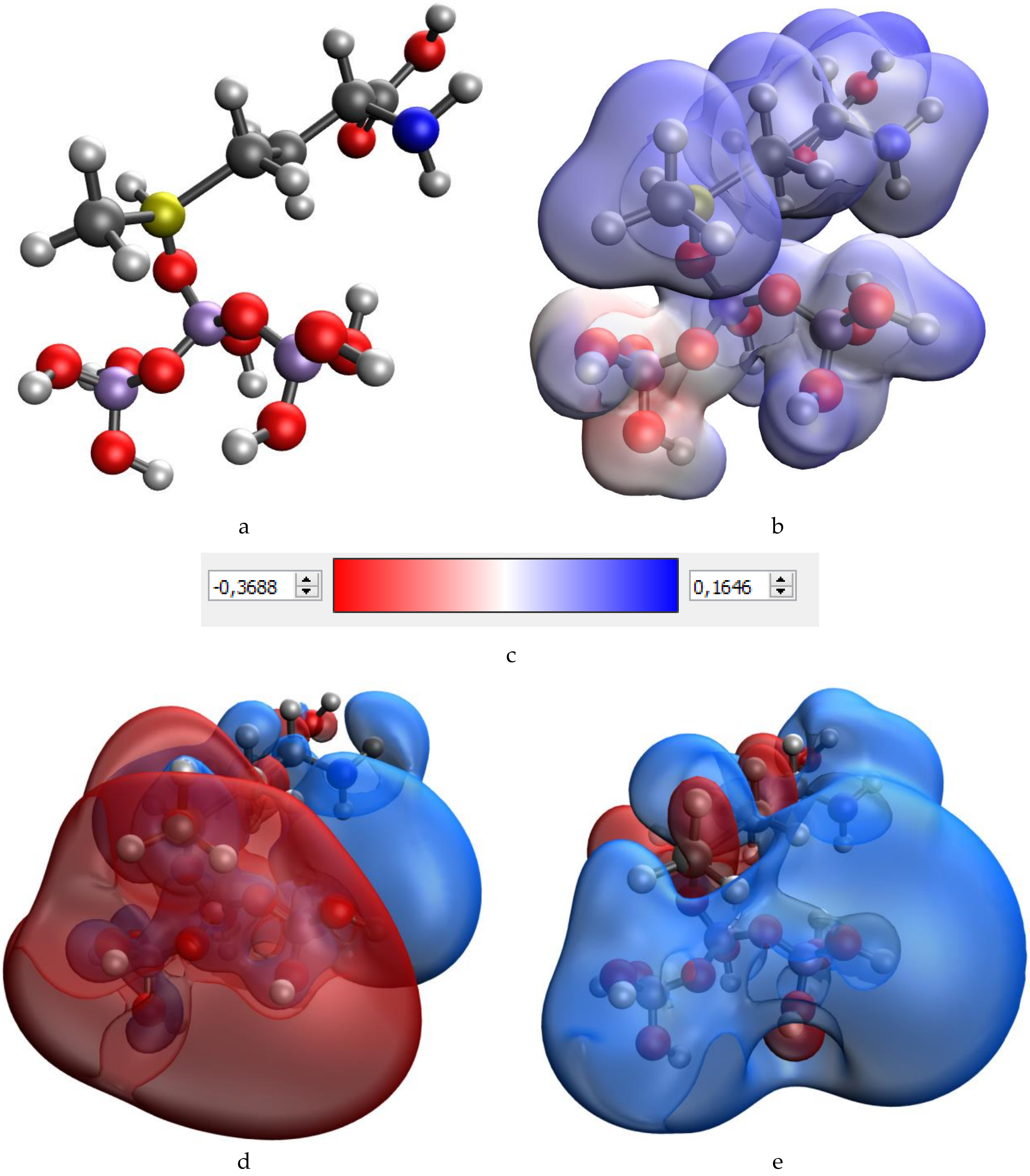

Figure S4. Interaction of the oxygen of the sulfo group of methionine sulfoxide and the hydroxo group of the middle manganese atom of MnO_2: model of the molecular complex (a), electron density distribution (b), electron density distribution gradient (c), HOMO molecular orbital (d), LUMO molecular orbital (e)

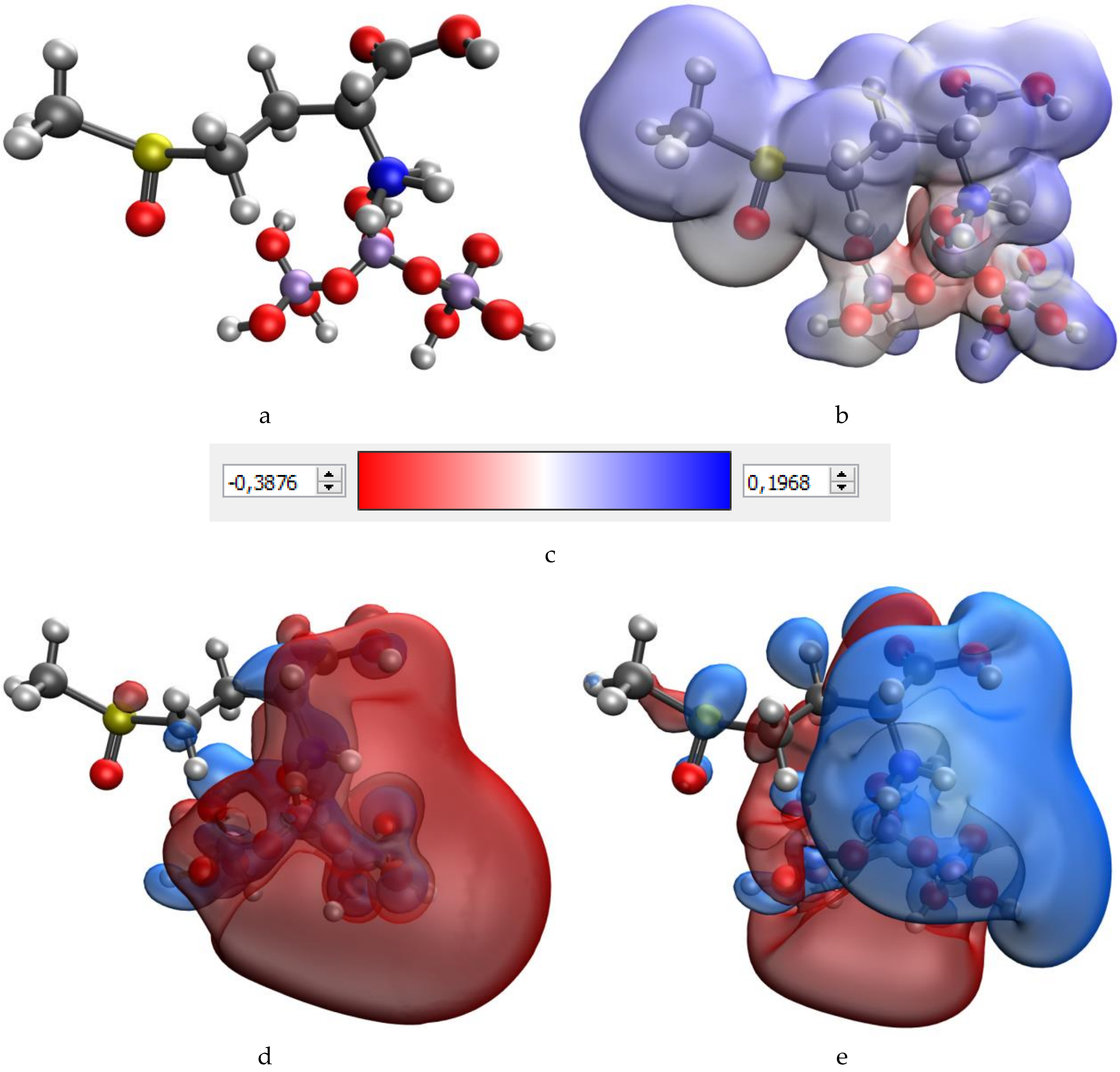

Figure S5. Interaction of the amino group of methionine sulfoxide and the hydroxo group of the middle manganese atom of MnO_2: model of the molecular complex (a), electron density distribution (b), electron density distribution gradient (c), HOMO molecular orbital (d), LUMO molecular orbital (e)

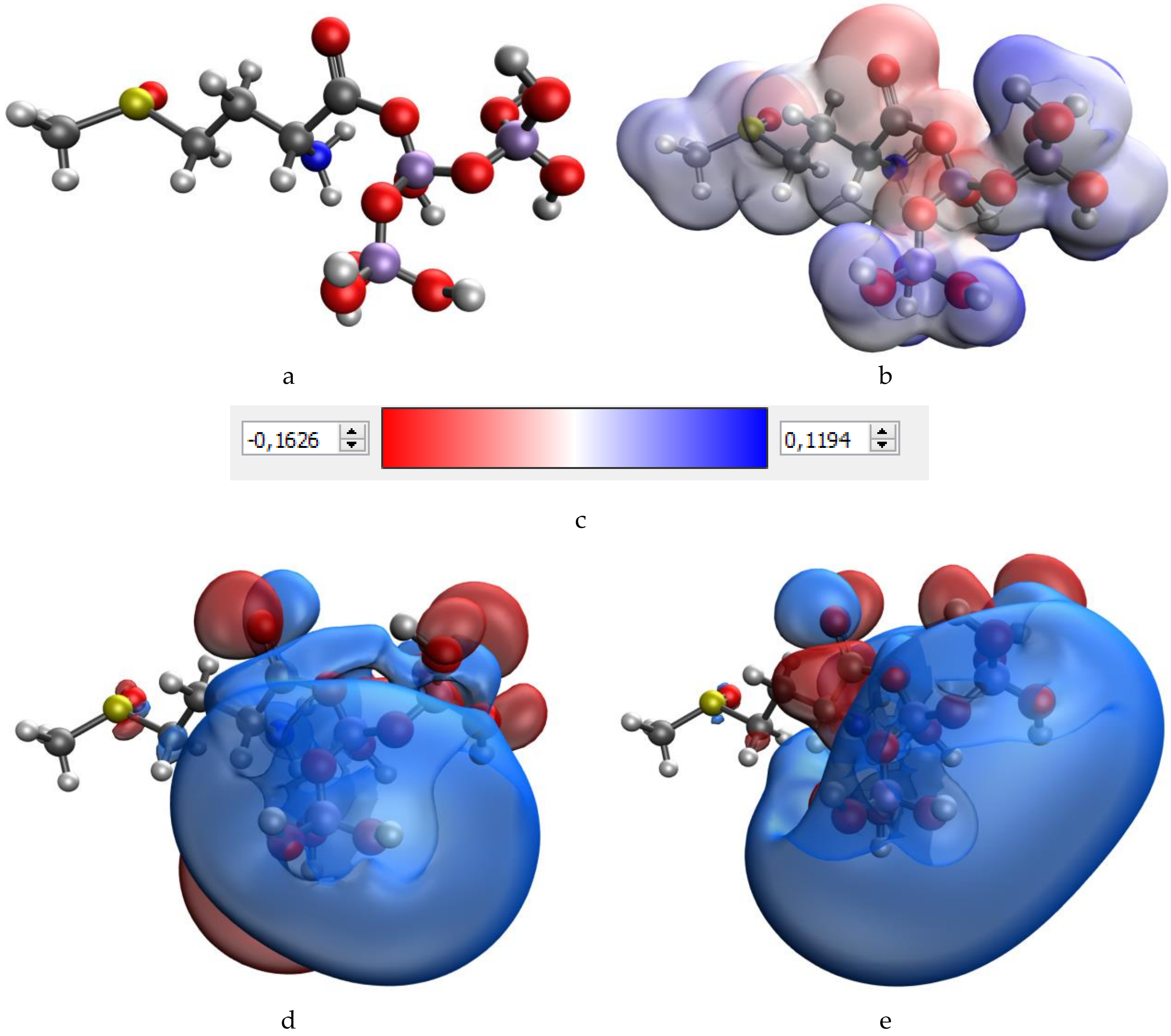

Figure S6. Interaction of the carboxyl group of methionine sulfoxide and the hydroxo group of the middle manganese atom of MnO_2: model of the molecular complex (a), electron density distribution (b), electron density distribution gradient (c), HOMO molecular orbital (d), LUMO molecular orbital (e)

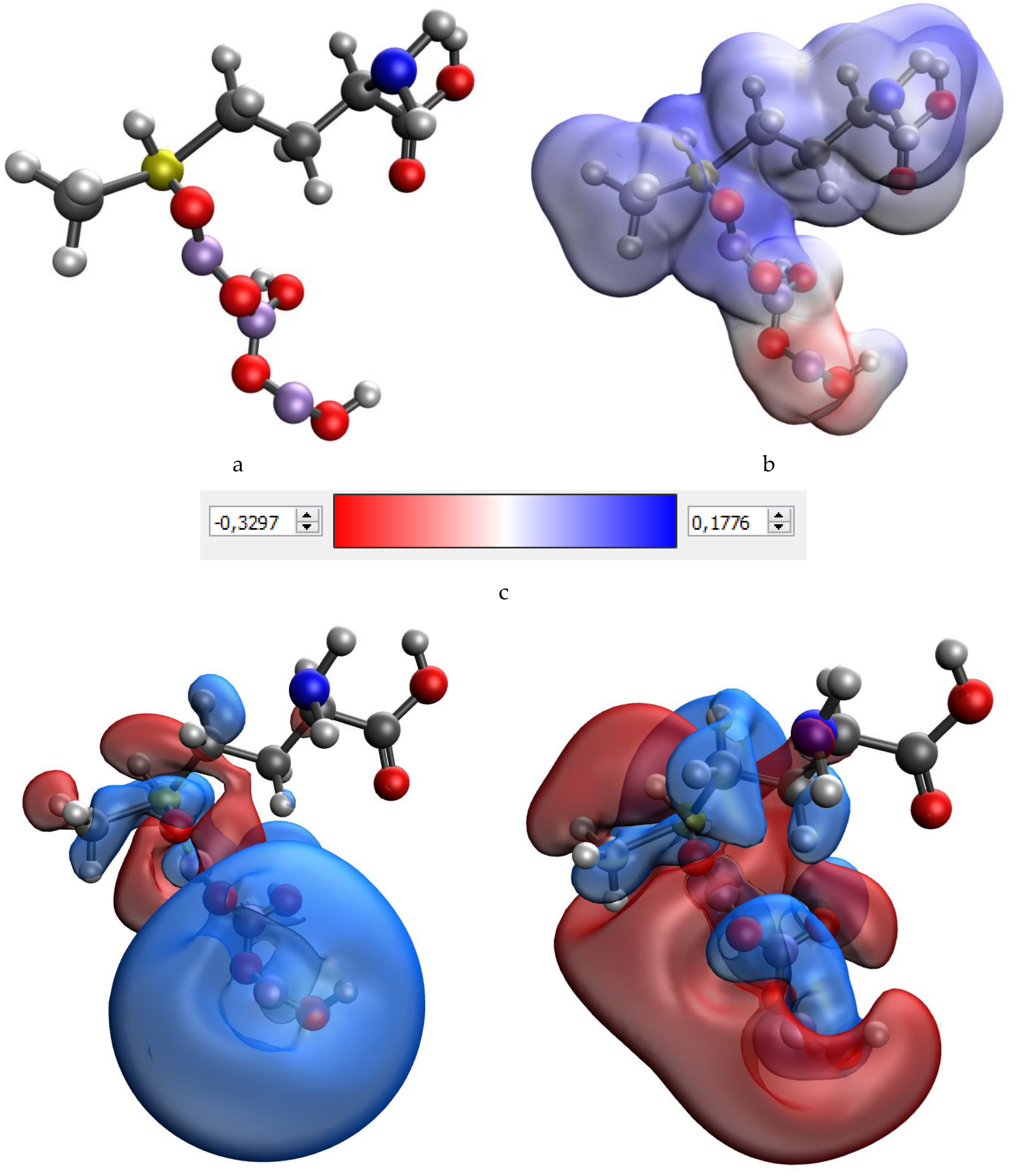

Figure S7. Interaction of the oxygen of the sulfo group of methionine sulfoxide and the hydroxo group of the extreme manganese atom of Mn_3O_4: model of the molecular complex (a), electron density distribution (b), electron density distribution gradient (c), HOMO molecular orbital (d), LUMO molecular orbital (e)

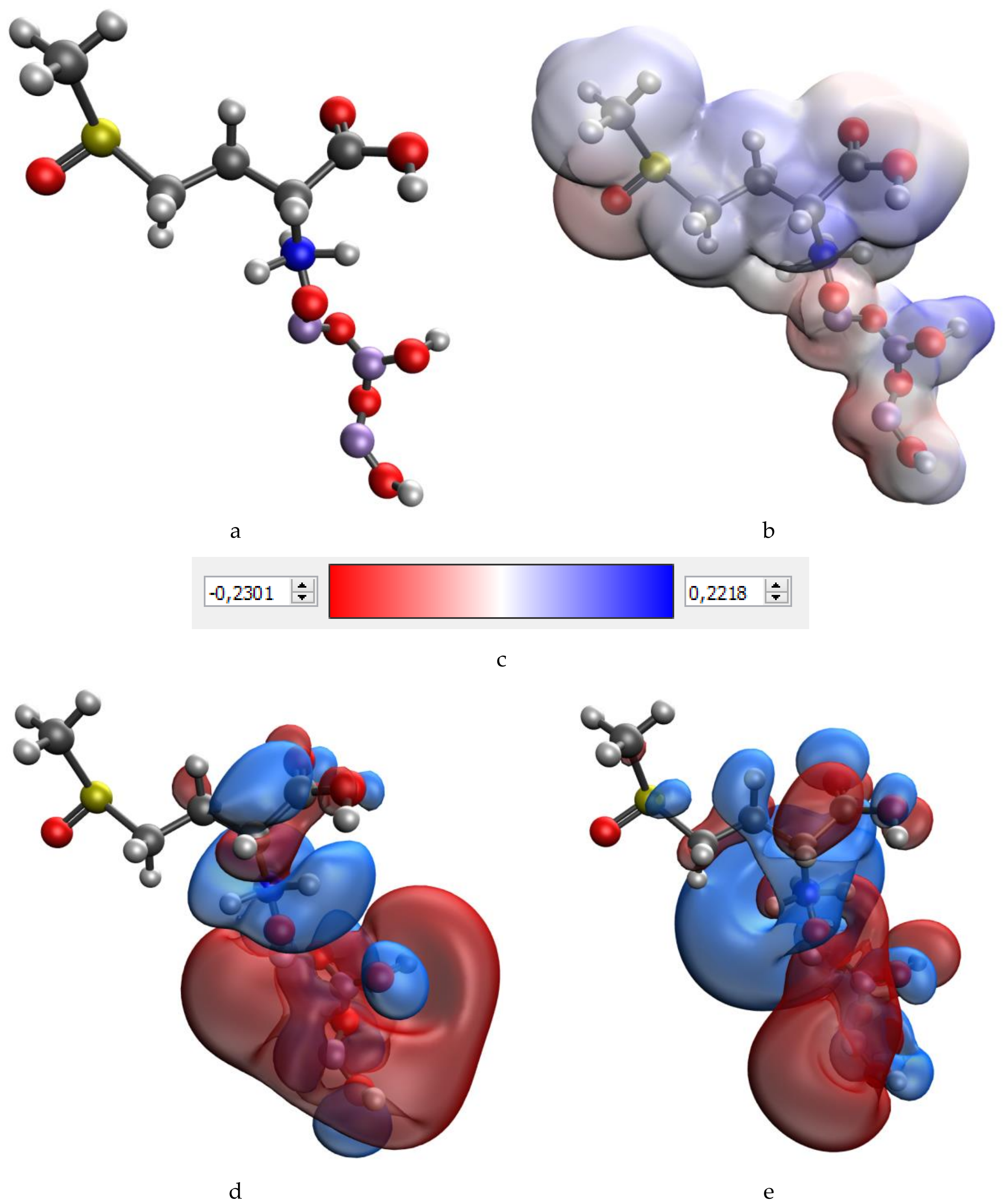

Figure S8. Interaction of the amino group of methionine sulfoxide and the hydroxo group of the extreme manganese atom of Mn_3O_4: model of the molecular complex (a), electron density distribution (b), electron density distribution gradient (c), HOMO molecular orbital (d), LUMO molecular orbital (e)

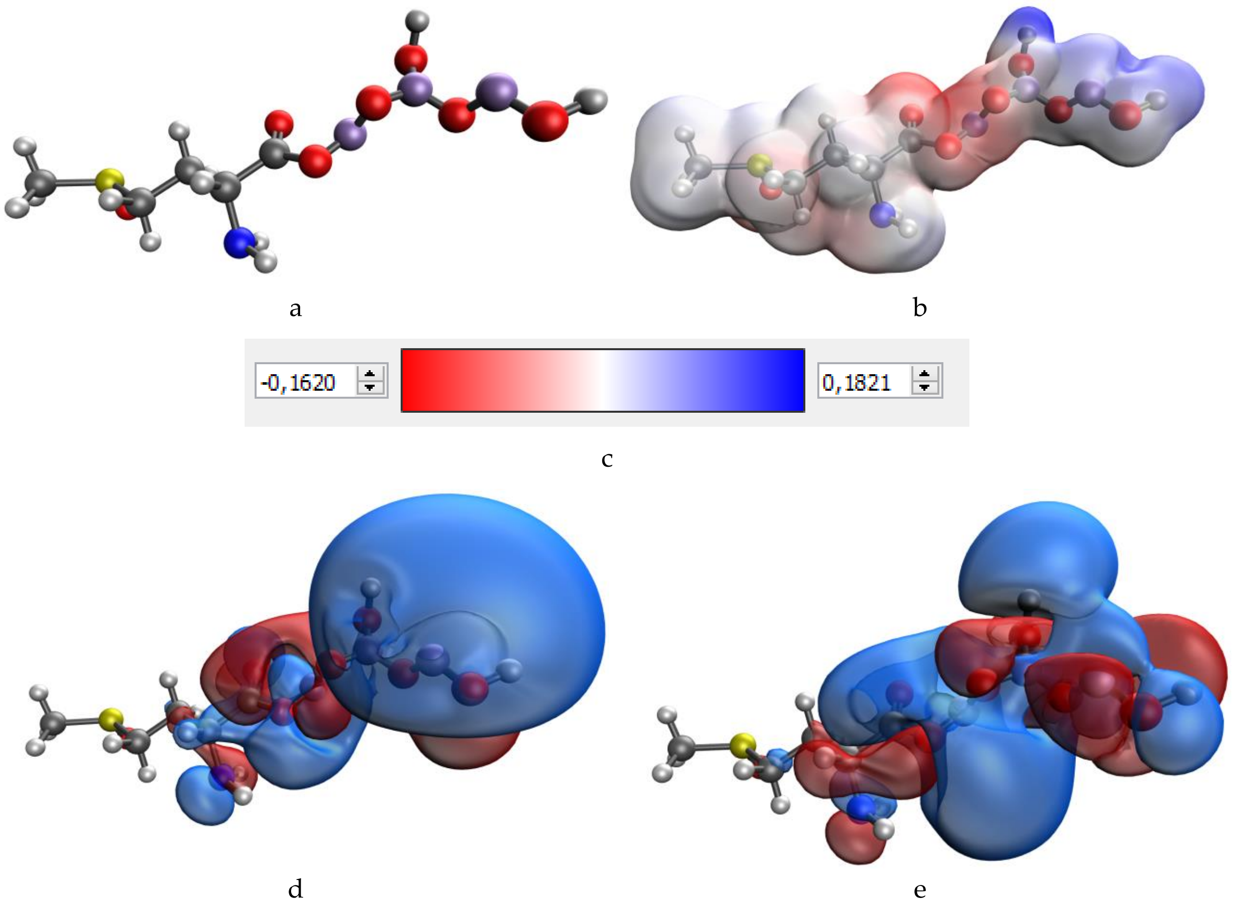

Figure S9. Interaction of the carboxyl group of methionine sulfoxide and the hydroxo group of the extreme manganese atom Mn_3O_4: model of the molecular complex (a), electron density distribution (b), electron density distribution gradient (c), HOMO molecular orbital (d), LUMO molecular orbital (e)

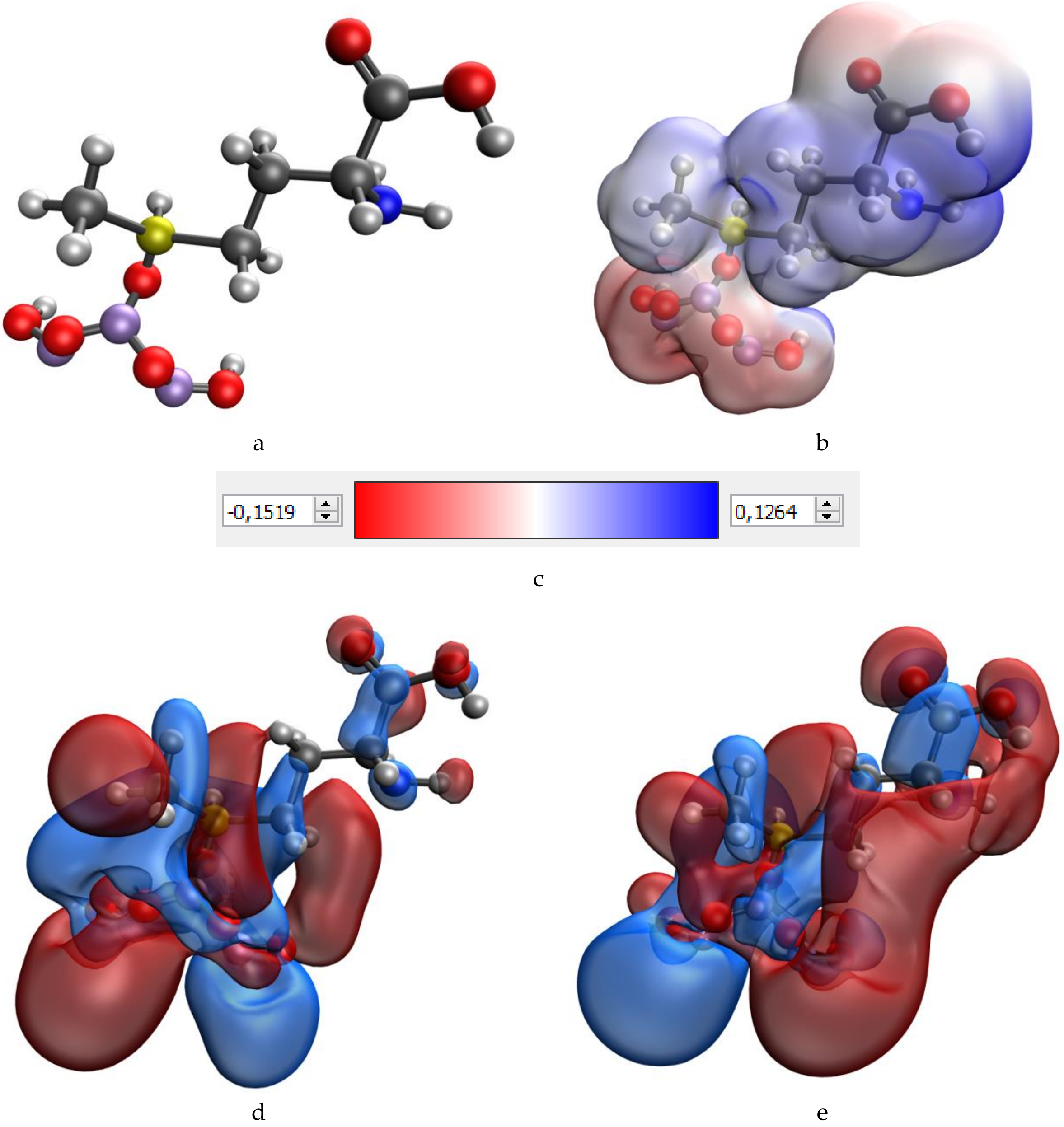

Figure S10. Interaction of the oxygen of the sulfogroup of methionine sulfoxide and the hydroxo group of the middle manganese atom of Mn_3O_4: model of the molecular complex (a), electron density distribution (b), electron density distribution gradient (c), HOMO molecular orbital (d), LUMO molecular orbital (e)

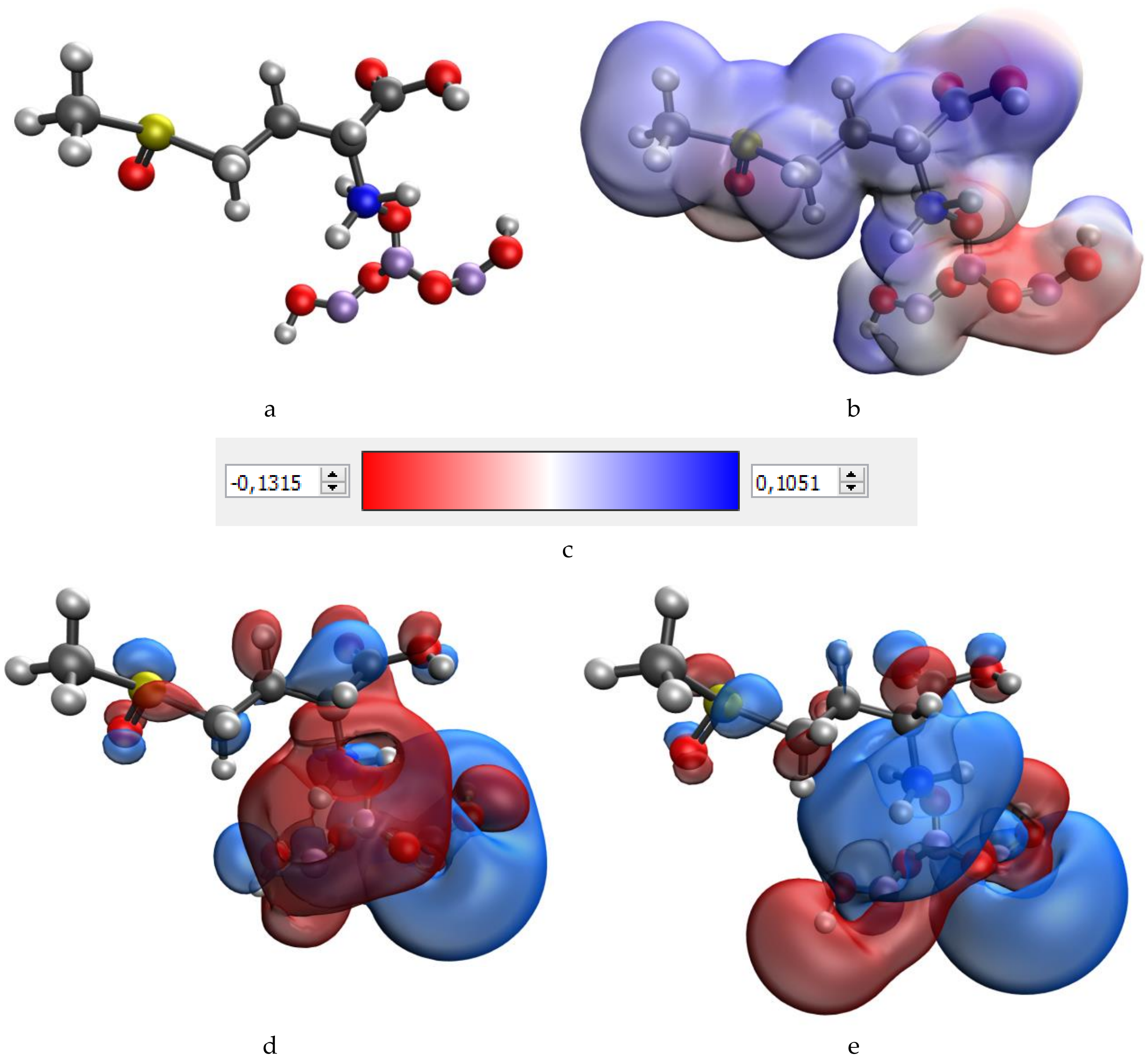

Figure S11. Interaction of the amino group of methionine sulfoxide and the hydroxo group of the middle manganese atom of Mn_3O_4: model of the molecular complex (a), electron density distribution (b), electron density distribution gradient (c), HOMO molecular orbital (d), LUMO molecular orbital (e)

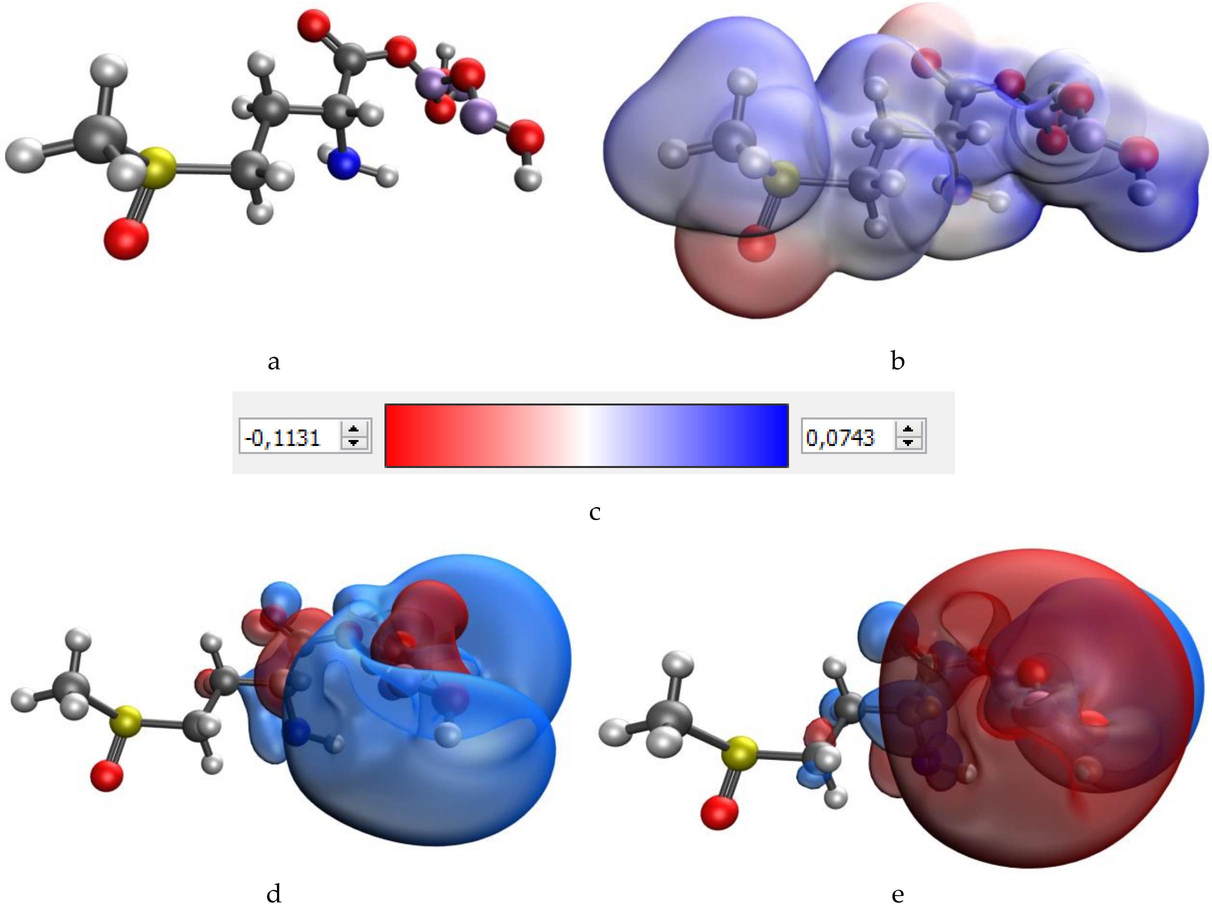

Figure S12. Interaction of the carboxyl group of methionine sulfoxide and the hydroxo group of the middle atom of Mn_3O_4: model of the molecular complex (a), electron density distribution (b), electron density distribution gradient (c), HOMO molecular orbital (d), LUMO molecular orbital (e)

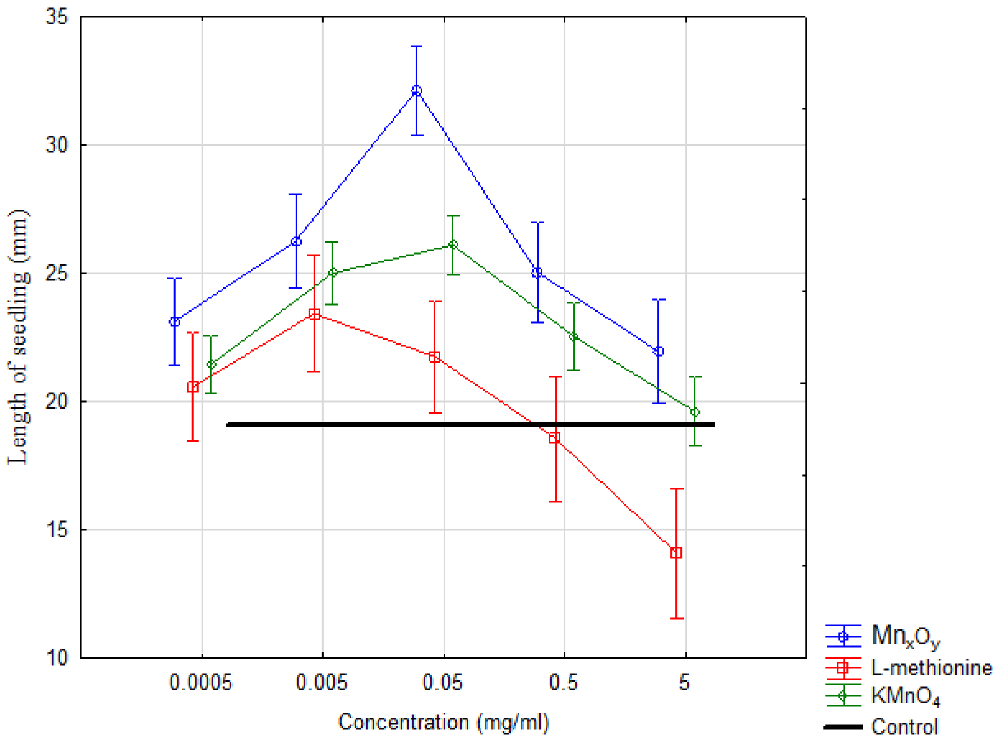

Figure S13. Dependence of distribution of length of seeding on concentration of substances (72 h incubation)

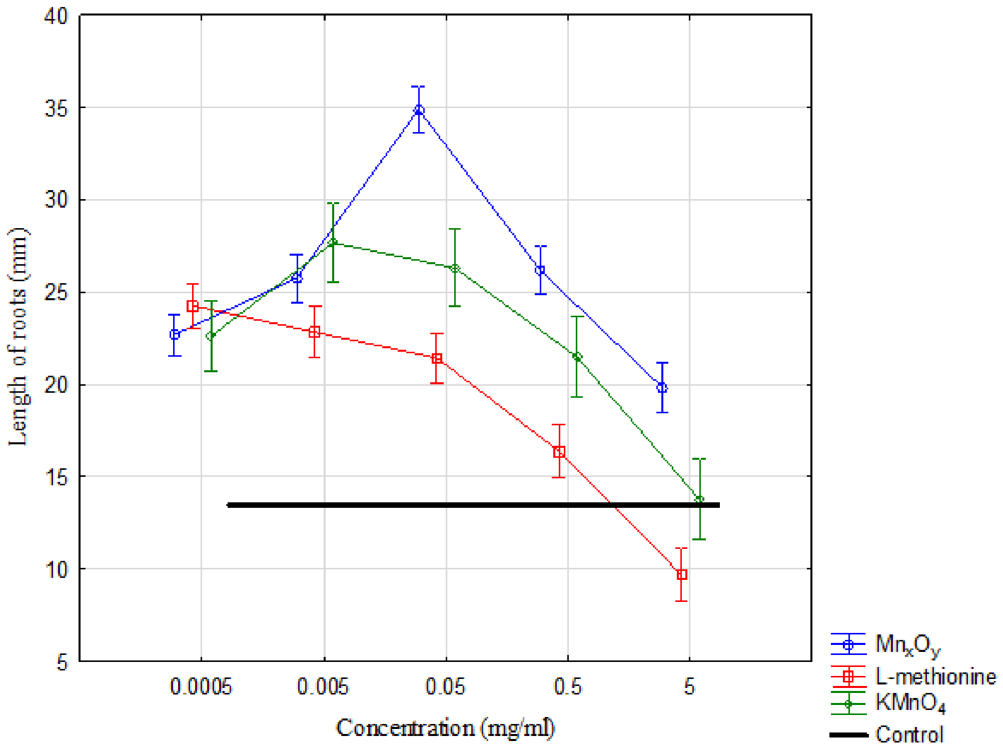

Figure S14. Dependence of length of roots on concentration of substances (72 h incubation)

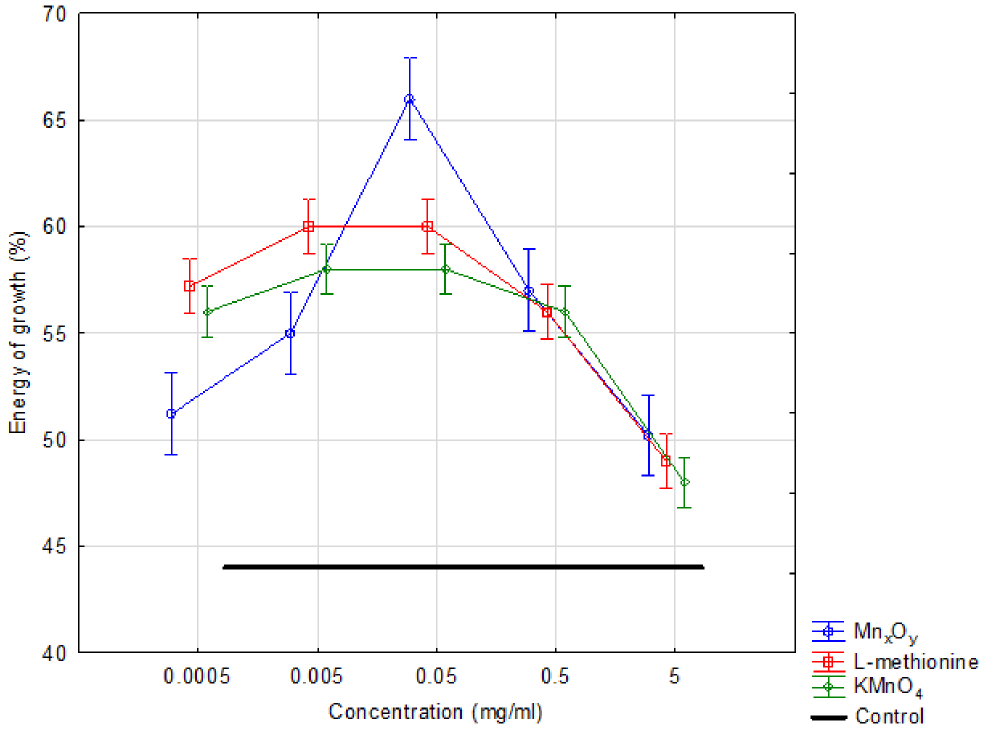

Figure S15. Dependence of distribution of germination energy on concentration of substances, (72 h incubation)
